# Risk Factor Assessments and Lipid Management Patterns in Hospitalised Patients in Malaysia: The Atherosclerotic Cardiovascular Disease Study

**DOI:** 10.21315/mjms-04-2025-294

**Published:** 2025-10-31

**Authors:** Azhari Rosman, Zhi Ling Looi, Mayuresh Fegade, Chan Ho Thum

**Affiliations:** 1 Cardiology Department, National Heart Institute, Kuala Lumpur, Malaysia; 2 Novartis Malaysia, Petaling Jaya, Selangor, Malaysia

**Keywords:** ASCVD, cardiovascular risk factors, hospitalised ASCVD patients, LDL-C goal, lipid-lowering therapy

## Abstract

**Background:**

Elevated levels of low-density lipoprotein cholesterol (LDL-C) result in dyslipidaemia, increasing the risk of atherosclerotic cardiovascular disease (ASCVD). As of 2022, Malaysian population (56.7%) demonstrated high LDL-C, necessitating an analysis of cardiovascular risk factors (CVRFs), comorbidities and treatment using lipid-lowering therapies (LLTs) to achieve target LDL-C < 1.4 mmol/L, as per Malaysian guidelines.

**Methods:**

This retrospective cohort study of 431 hospitalised patients (≥ 18 years) with elevated LDL-C levels from the National Heart Institute, Malaysia, assessed CVRFs, comorbidities, treatment patterns, and the proportion of patients achieving LDL-C goals (< 1.8 mmol/L, < 1.4 mmol/L, and < 1.0 mmol/L) after LLT. The association pattern of CVRFs was studied in patients achieving the target goal of < 1.4 mmol/L.

**Results:**

The mean age of the patients was 60.53 (9.86) years, with the majority being male (79.35%), non-smokers (84.78%), and Malay (61.48%). Most patients (88.40%) had acute coronary syndrome, and the most prevalent risk factors were systemic hypertension (76.33%), diabetes mellitus (DM) (51.97%), and chronic kidney disease (CKD) (28.54%). Monotherapy with high-intensity statins was the most common LLT. At 1 year, 48.26%, 24.83% and 7.66% of patients had achieved target goals of < 1.8 mmol/L, < 1.4 mmol/L, and < 1.0 mmol/L, respectively. Poor LDL-C achievement was observed among patients with DM and recurrent events (*P* = 0.0097), left ventricular ejection fraction < 35% (*P* = 0.0325), and age > 75 years (*P* = 0.0087).

**Conclusion:**

Hypertension, diabetes and CKD are prevalent risk factors, and despite high-intensity statins, only 24.83% of patients achieved the LDL-C target goal. Thus, the use of combination therapy and innovative therapies is necessary to improve ASCVD management in Malaysia.

## Introduction

Cardiovascular disease (CVD) is the leading cause of mortality and disability in the world. As per the World Health Report 2023, CVD continues to impact over half a billion people globally and was responsible for 20.5 million deaths in 2021, nearly one-third of all deaths worldwide (1). Consistently, CVD is one of the major non-communicable diseases (NCDs) leading to 33.7% and 33.1% of mortality among men and women respectively, in Malaysia (2). Hypertension, diabetes and dyslipidemia are the most common cardiovascular risk factors (CVRFs) observed in patients with acute coronary syndrome (ACS) (3). Additionally, obesity, chronic kidney disease (CKD), poor dietary habits, physical inactivity, smoking and psychosocial stress are other risk factors for atherosclerotic cardiovascular disease (ASCVD) (4, 5). As per the Malaysian National Health and Morbidity Survey 2023 (6), 2.5% of the Malaysian adults live with four NCDs, namely hypertension, high cholesterol, diabetes, and obesity, which are major risk factors for heart disease and stroke. Approximately 7.6 million adults have high cholesterol, with 41% having high LDL, 27% having low HDL and 23% having high triglyceride levels (6). Atherosclerosis or plaque buildup of fatty and/or fibrous substances within the inner layer of arteries, results in narrowing of the arteries’ diameter, leading to coronary heart disease (CHD), stroke and peripheral arterial disease (PAD) (7, 8). It is a major cause of fatalities and medical conditions around the globe and is expected to be the leading cause of mortality by 2030 (9). Dyslipidemia, characterised by elevated low-density lipoprotein cholesterol (LDL-C), increases ASCVD risk (10). As of 2022, in Malaysia, 56.7% and 64% of the total population have increased LDL-C and total cholesterol (TC), respectively (11, 12).

Elevated LDL-C levels, a causal factor in ASCVD, are a primary target of dyslipidaemia management (13, 14). According to the 2023 Malaysian clinical practice guidelines (CPG), LDL-C reduction of >50% from baseline and an LDL-C goal of ≤1.8 mmol/L are recommended for high-risk patients. For very high-risk patients, LDL-C reduction of > 50% from baseline and an LDL-C goal of ≤ 1.4 mmol/L are recommended. Furthermore, for ASCVD patients experiencing a second vascular event (within two years) despite achieving the < 1.4 mmol/L target goal, an LDL-C goal of < 1.0 mmol/L is recommended (15). The 2019 European Society of Cardiology/European Atherosclerosis Society (ESC/EAS) guidelines and the 2023 ESC Guidelines for the Management of ACS recommend similar treatment goals in patients with risk of CVD (16, 17). The risk of ASCVD is reduced by using lipid-lowering therapies (LLTs) such as (18) statin therapy which can be used in combination with cholesterol absorption inhibitors (ezetimibe), proprotein convertase subtilisin/kexin type 9 (PCSK9) targeted therapy [such as PCSK9 inhibitors (PCSK9i) or small interfering RNA (siRNA) therapy] (19) depending upon ASCVD risk and lipid levels, according to ESC/EAS guidelines (16, 18).

Residual risk is still reported despite vigorous intervention with statins, specifically in patients having diabetes mellitus (DM) (20, 21), obesity and metabolic syndrome (22). Therefore, in addition to statins, more treatment with alternative and novel therapies may be required (22). A disparity exists between guidelines recommended LLT usage and real-world clinical practice (23). Moreover, patient adherence to LLTs should be improved to achieve LDL-C goals better. Therefore, further evaluations are needed to optimise lipid management and achieve targeted LDL-C goals using LLT by improving patient adherence (24, 25). Currently, data on outcomes among Malaysian patients with ASCVD are limited. Therefore, a retrospective, non-interventional, observational, single-centre, cohort study [Atherosclerotic Cardiovascular Disease (AcarD)] was conducted to evaluate the risk factors, comorbidities, LDL-C attainment, and LLT patterns to achieve the target LDL-C goal (< 1.4 mmol/L) in hospitalised ASCVD patients.

## Methods

### Study Design

This is a single-centre, non-interventional and retrospective cohort study of adult patients with ASCVD and dyslipidaemia, conducted between 1 January 2020 and 28 February 2021 using medical records from the National Heart Institute, Malaysia [Institut Jantung Negara (IJN)]. Data from 431 patients with elevated LDL-C levels who were hospitalised in the cardiology ward and the intensive care unit were collected. The baseline index date was defined as the first hospital admission associated with ASCVD diagnosis and LLT treatment in this study.

The study was designed, implemented, and reported in accordance with the Good Pharmacoepidemiology Practices guidelines of the International Society for Pharmacoepidemiology (ISPE 2016), the Strengthening the Reporting of Observational Studies in Epidemiology (STROBE) guidelines (26) and the Declaration of Helsinki. The study was approved by the IJN Research Ethics Committee (registration number: IJNREC/552/202), met the criteria of the European Network of Centres for Pharmacoepidemiology and Pharmacovigilance (ENCePP) study, followed the ENCePP Code of Conduct, and is based on the approved protocol (CKJX839A1MY02R). Target LDL-C levels were based on the patient’s CVD risk factors.

### Inclusion and Exclusion Criteria

**Figure 1:**
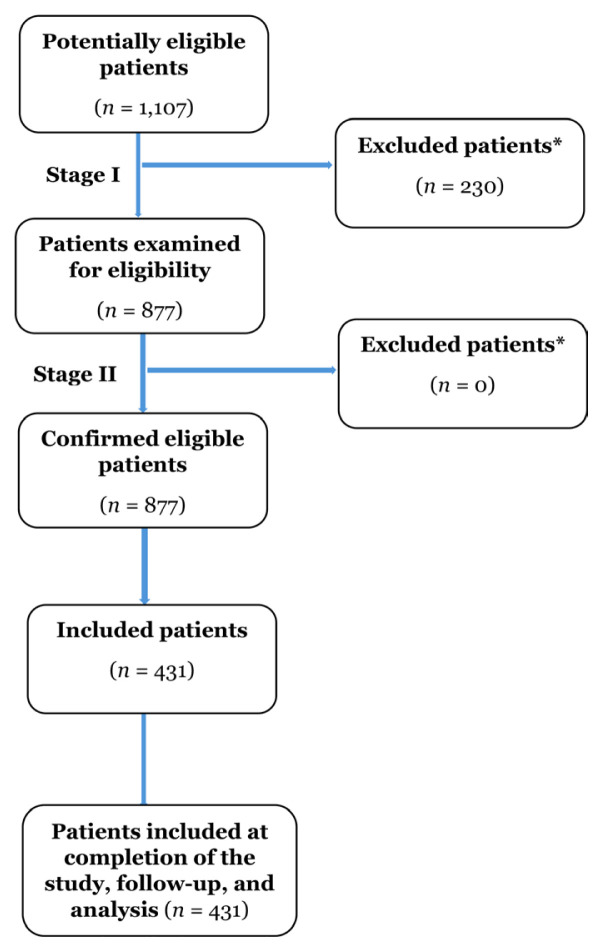
Participants flowchart *Reason for non-participation of patient at any-stage: Absence of patient at 1 year follow-up

Patients aged ≥ 18 years, hospitalised due to ASCVD during the study period and with dyslipidaemia were included. Patients who participated in other clinical trials or with clinical pregnancy at the index date were excluded from the study. The process for patient inclusion is depicted in Figure 1.

### Variables

The risk factors/comorbidities included hypertension, DM, CKD, the occurrence of a recurrent episode of ASCVD-related events in < 12 months, DM with recurrent events, smoking, body mass index (BMI) (kg/m^2^), multivessel coronary artery disease (CAD), PAD, history of cardiac intervention, young age at first ASCVD event (< 40 years), age > 75 years, left ventricular ejection fraction (LVEF) < 35% and atrial fibrillations (AF). Data on LLT patterns such as statins, ezetimibe, PCSK9i, a combination of statins and ezetimibe, and other LLT drugs were recorded. The baseline variables included age, gender, race, smoking history, BMI, LDL-C, TC, and estimated glomerular filtration rate (eGFR). As per the Malaysian CPG for the Management of Obesity 2023, the BMI range was classified as underweight (< 1.85 kg/m^2^), normal (18.5 to 22.9 kg/m^2^), overweight (23 to 27.4 kg/m^2^), and obese (≥ 27.5 kg/m^2^) (27).

The clinical variables included LDL-C level (mmol/L), TC (mmol/L), eGFR (%) and clinical ASCVD [such as ACS, angina, PAD, ischaemic stroke and transient ischaemic disease (TIA)] and vital signs [heart rate (bpm) and blood pressure (mmHg)]. For the ASCVD patients with a history of ≥ 1 CV event (ACS, angina, PAD, ischaemic stroke or TIA), the number of prior hospital admissions within 1 year ± 60 days of the last hospitalisation was evaluated.

### Study Endpoints

The primary endpoint was to assess the proportion of CVRFs or comorbidities among the ASCVD patients.

The secondary endpoints were to evaluate the treatment patterns at baseline, 6 months, and 1 year. Further, the demographic and clinical characteristics of the patients were studied at both baseline and 1 year.

The exploratory endpoint was to analyse the proportion of patients who attained LDL-C goals of < 1.8 mmol/L, < 1.4 mmol/L, and < 1.0 mmol/L, stratified by LLT type and key patient subgroups. Further, the goal attainment of < 1.4 mmol/L data was stratified by LLT and key patient subgroups (based on the presence or absence of risk factors).

### Statistical Analysis

Sample size calculation was performed with nMaster (v2.0) software, using the single–proportion method to estimate the population proportion with absolute precision. A minimum of 385 patients was required, considering that 50.8% of patients with ASCVD were at high-risk (28). The assumed precision was within 5% of the given proportion, with a 95% confidence interval. The statistical analysis was performed using Statistical Package for Social Sciences (SPSS) v23.0 software. Patients were classified as per the risk factor categories, and risk factors were tabulated along with ASCVD patterns for the primary objective.

For the secondary objectives, LLTs were analysed along with the ASCVD patterns. Further, all demographic and clinical characteristics of the main study cohort were analysed as one of the secondary endpoints. Mean/median and standard deviation (SD) were used to summarise all continuous, demographic, and clinical variables.

The proportion of patients achieving the LDL-C target goal, stratified by LLT patterns, was analysed using the *P*-value and Chi-square tests to study the association between the proportion of patients achieving the LDL-C target goal (< 1.4 mmol/L) and LLT patterns. Additionally, *P*-values and Chi-square were used to evaluate the correlation between CV risk categories and LDL-C target goal achievement (< 1.4 mmol/L). The exploratory objectives were also classified into categories and cross-tabulated.

All endpoints were counted, expressed as percentages and represented as a summary. Statistical significance was considered for a *P*-value < 0.05.

## Results

### Patient Characteristics at Baseline

The baseline characteristics of 431 ASCVD patients are shown in Table 1. The majority of patients in the AcarD study were male (79.35%), and the mean (SD) age at admission was 60.53 (9.86) years, with 49.88% of patients aged 60 to 75 years. The majority of the study population were Malays (61.48%), and most of the study patients were non-smokers (84.78%) (Table 1). The mean (SD) baseline BMI for the patients was 27.79 (4.53). Further, the patients’ mean (SD) body weight at admission was 74.09 (14.58) kg.

At baseline, the mean (SD) LDL-C was 1.97 (0.88) mmol/L, and the majority [33.64% (145/431)] of the patients had LDL-C levels between 1.8 and 2.59 mmol/L. The mean (SD) TC at baseline was 3.74 (0.97) mmol/L. In addition, the mean (SD) heart rate was 69.84 (11.74) bpm with a median of 70 bpm. For systolic blood pressure (BP), the mean (SD) was 134.82 (20.74) mmHg, and the mean (SD) diastolic BP was 77.16 (11.46) mmHg. The proportion of ASCVD patients with a history of ≥ 1 CV event (ACS, angina, PAD, ischaemic stroke, or TIA) was 13.69% (59/431). Further, 15.31% (66/431) of patients were re-hospitalised within 1 year ± 60 days of the last hospitalisation in this study.

### ASCVD Profile and Associated Risk Factors

Analysis of the clinical ASCVD types revealed that 88.40% (381/431) of patients had ACS [myocardial infarction (MI) or unstable angina (UA)], 23.43% (101/431) had stable angina, 1.16% (5/431) had PAD, 0.23% (1/431) had ischaemic stroke and 0.23% (1/431) of patients had TIA.

The most prevalent risk factor/co-morbidity among the ASCVD population with dyslipidaemia was systemic hypertension (76.33%), followed by DM (51.97%) and CKD (28.54%) at baseline. Elevated BMI emerged as another risk factor, with a predominant number of individuals categorised as overweight (46.22%) and obese (27.13%). The proportion of patients stratified by various risk factors is further described in Table 2.

### LLT Patterns

**Figure 2:**
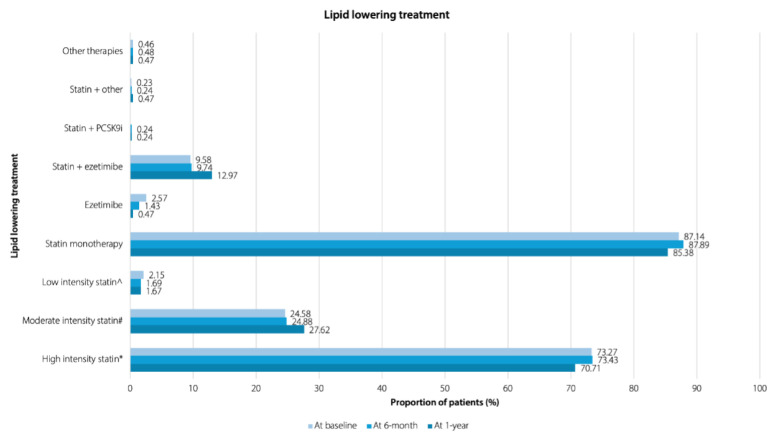
Proportion of patients on one or more LLTs at baseline, 6 months and 1 year PCSK9 = proprotein convertase subtilisin/kexin type 9; *Atorvastatin 40 mg to 80 mg/Rosuvastatin 20 mg to 40 mg; #Atorvastatin 10 mg to 20 mg/Rosuvastatin 5 mg to 10 mg/Simvastatin 20 mg, etc.; ^Pravastatin 10 mg to 20 mg/Simvastatin 10 mg, etc.

Patients in this study were treated with LLTs such as statins, ezetimibe, PCSK9i, or combination treatments (statins + ezetimibe, statins + PCSK9i). From baseline to 1-year, statin monotherapy (85.38% to 87.89%) was the most commonly administered LLT, with the majority of the patients (70.71% to 73.43%) undergoing treatment with high-intensity statins, followed by moderate-intensity (24.58% to 27.62%), and low-intensity (1.67% to 2.15%) statins (Figure 2).

### Attainment of Guideline-recommended LDL-C Goals

**Figure 3:**
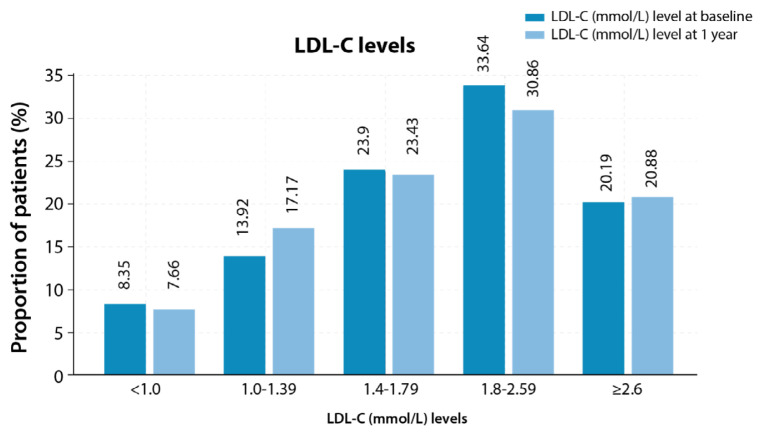
The LDL-C (mmol/L) levels at baseline and 1 year (categorical) LDL-C = low-density lipoprotein cholesterol

**Figure 4:**
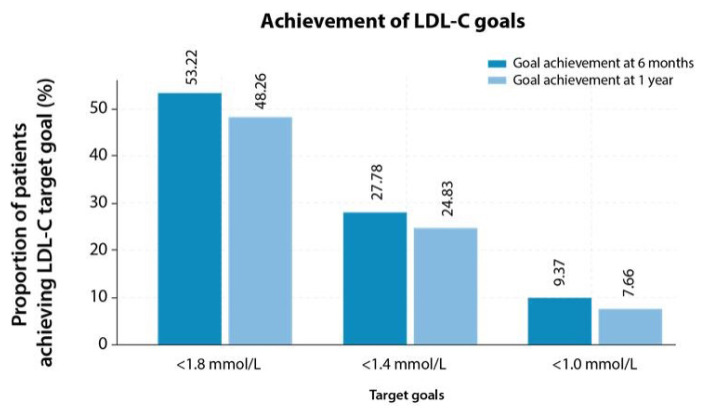
Proportion of patients achieving target goals after treatment with LLTs LDL-C = low-density lipoprotein cholesterol; LLT = lipid-lowering therapy

The majority of the patients (33.64% and 30.86%) had LDL-C levels between 1.8 and 2.59 mmol/L at baseline and 1 year, respectively (Figure 3). At 1 year, 48.26% (208/431) of patients achieved < 1.8 mmol/L, 24.83% (107/431) achieved the LDL-C target of < 1.4 mmol/L, and 7.66% (33/431) achieved the target goal of < 1.0 mmol/L (Figure 4). Out of 105 patients who achieved the LDL-C target goal of < 1.4 mmol/L (there was no treatment data for two patients at 1 year), 83.81% (88/105) of patients were on statin monotherapy, 1.90% (2/105) of patients were on ezetimibe and 13.33% (14/105) of patients were administered with a combination of statins + ezetimibe. Furthermore, 0.95% (1/105) of patients were treated with statin + PCSK9i.

Among 370 patients administered statin monotherapy, 23.78% (88/370) achieved the desired target LDL-C goal (< 1.4 mmol/L) at 1 year. Furthermore, 33.33% (2/6) and 34.14% (14/41) of patients who were on ezetimibe and statin + ezetimibe therapy, respectively, attained the treatment goal at 1 year. Additionally, one patient treated with statin + PCSK9i achieved the target goal of < 1.4 mmol/L.

### LDL-C Goal Attainment Stratified by Key Patient Subgroups

The association pattern of CVRFs was evaluated among patients at the target goal of < 1.4 mmol/L in the exploratory endpoint.

**Figure 5:**
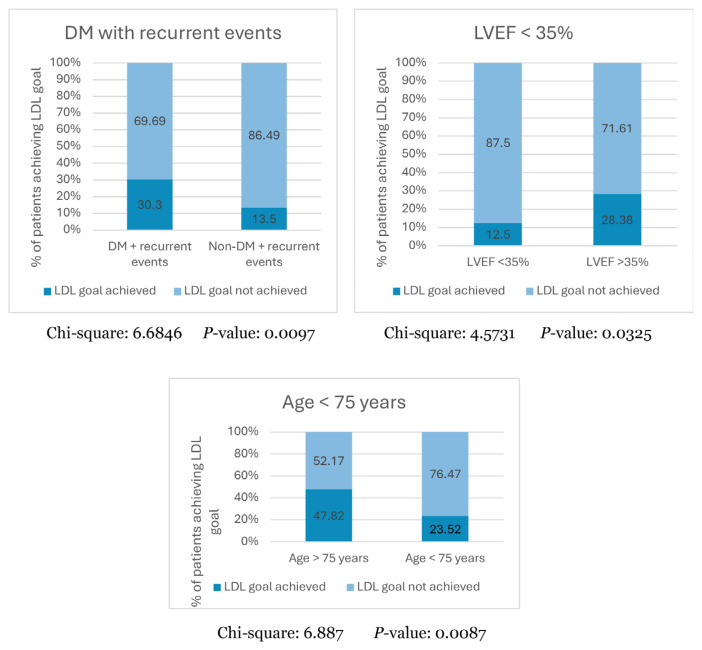
LDL-C goal attainment stratified by key patient subgroups DM = diabetes mellitus; LDL-C = low-density lipoprotein cholesterol; LVEF = left ventricular ejection fraction; NA = not available

In patients having DM with recurrent events, 30.3% of the patients could achieve the LDL-C goal of < 1.4 mmol/ L; however, only 13.5% achieved the LDL-C goal in patients without DM and recurrent events (*P* = 0.0097). Further, among patients with LVEF < 35%, only 12.5% attained the LDL-C goal of < 1.4 mmol/L, compared with 28.38% of patients with LVEF > 35% who achieved the target (*P* = 0.0325). Moreover, in patients aged > 75 years, higher proportion of patients (47.82%) could achieve the target goal in comparison to patients < 75 years of age (23.52%) (*P* = 0.0087) (Figure 5).

Additionally, other risk factors such as DM, recurrent episodes of ASCVD, CKD, elevated BMI, smoking, hypertension, AF, multivessel CAD, history of cardiac surgery/intervention, and patients < 40 years were evaluated and had no significant correlation in achieving the LDL-C target goal of < 1.4 mmol/L.

## Discussion

Data from the AcarD study conducted at the IJN demonstrate the prevalence of ASCVD-related risk factors and lipid management patterns among hospitalised ASCVD patients. Risk factors such as age, hypercholesterolaemia, hypertension, DM and obesity contribute to the development of CVD (16, 29). In the AcarD study, hypertension (76.33%) was the dominant risk factor among the very high-risk ASCVD patients with dyslipidaemia, and most of the patients were overweight (45.48%) or obese (26.68%). Similarly, in a UAE-based retrospective study, hypertension was observed in 94.9% of the study population, while 38.4% and 27.3% of the ASCVD patients were overweight and obese, respectively (30). Govender et al. (30) reported DM, present in a very high proportion (62.5%), to be the second-highest-risk factor and one of the significant risk predictors of recurrent CVD events or death in CVD patients. Similarly, the second most common risk factor in the AcarD study was DM (51.97%). As per the National Cardiovascular Disease Database–Percutaneous Coronary Interventions (NCVD–PCI) registry, 44.0% of Malaysians with PCI had diabetes, 54.8% had dyslipidaemia, and were younger compared to Japanese patients (31). Additionally, the NCVD-ACS registry reported dyslipidaemia in 36.1% of the Malaysian population, with better-controlled hypercholesterolaemia in the dyslipidaemic group than in the non-dyslipidaemic group (3). Moreover, between 2018 and 2019, 93.5% of ACS patients also exhibited other CVRFs such as diabetes (44.2%) and hypertension (61.9%), among others (3). In the AcarD study population, ACS was highly prevalent (88.40%), with diabetes (51.97%) and hypertension (76.33%) being the dominant risk factors.

At the end of 1 year, statin monotherapy was the most frequently prescribed LLT [85.38%, (362/424)] in the AcarD study, with predominant patients on high-intensity statins, whereas combination therapy was administered to 13.68% (58/424) of our patients. In Malaysia, high-intensity statin is the recommended treatment for patients with CHD or ACS before undergoing PCI and coronary artery bypass graft (CABG) (32). In the EU-wide DA VINCI study, the LDL-C goals (< 1.8 mmol/L and < 1.4 mmol/L) were achieved by 54% and 21% of the very high-risk patients, respectively. Statin monotherapy (83.3%) with high- and moderate-intensity statins is the most prescribed LLT compared to low-intensity statins among ASCVD patients (33). Combination therapy with ezetimibe or PCSK9i was administered to only 10.4% of the overall population in the DA VINCI study (33). Treatment adherence (34), side effects and drug-drug interactions are some of the limitations still associated with statins (35).

However, despite treatment with moderate (27.62%) and high-intensity (70.71%) statins in a high proportion of patients in the AcarD study at the end of 1 year, only 27.78% and 24.83% of patients achieved the LDL-C goal of < 1.4 mmol/L at 6 months and at 1 year, respectively. Further, at 6 months and 1 year, 53.22% and 48.26% of patients achieved the LDL-C goal of < 1.8 mmol/L, respectively. Approximately the same proportions of patients, achieving the LDL-C goal at 6 months and 1 year, could be the result of similar treatment regimens at both times in this study. Moreover, despite the optimal treatment with a statin, the gap between the LDL-C goals recommended in the guidelines and real-world clinical care necessitates increased use of non-statin combinations for the treatment of the highest-risk patients. Challenges with statins and the identification of PCSK9 led to the development of novel lipid-directed therapies, such as PCSK9i (alirocumab, evolocumab) and siRNA (inclisiran), which can reduce LDL-C levels by approximately 50% to 60% on top of standard therapy (19, 34, 36). Reduction in major CV events by intensifying the treatment with statins, ezetimibe and a combination of statin + ezetimibe has been reported in a study by Farnier et al. (37). To lower CV risk, it is essential to reduce LDL-C levels with targeted therapy (16). Elevated LDL-C over time can increase the total atherosclerotic burden by accumulating in the arterial wall, causing atherosclerotic plaque, which may eventually result in UA, MI or death (13). Reduction in LDL-C, an important modifiable risk factor, reduces the risk of ASCVD events, making it a treatment target in CVD patients (14). The 2023 Malaysian CPG on the Management of Dyslipidaemia recommends stringent treatment goals of ≤ 1.4 mmol/L and ≤ 1.8 mmol/L, with a > 50% reduction from baseline, for very high- and high-risk patients, respectively (15). Further, a target goal of < 1.0 mmol/L is recommended for patients at extremely high-risk who experience a second vascular event within 2 years despite achieving the < 1.4 mmol/L goal (15, 38). This is in line with the recommendations of other guidelines, namely, the 2019 ESC/EAS guideline (16), the 2023 ESC Guideline for the Management of ACS (17), the 2020 Malaysian Management of Type 2 Diabetes Mellitus (T2DM) CPG (39) and the 2021 Malaysian Non-ST Elevation ACS (NSTE-ACS) CPG (40). Additionally, the CV risk prediction models (such as the Framingham risk score) play a vital role in preventing, assessing, and managing CVDs (41). The combination of risk stratification of patients and treatments helps personalise the prevention therapies and facilitates the patient and clinician discussion (42).

Further, in the AcarD study, a significant association was observed between the occurrence of DM with recurrent events and LDL-C goal attainment (*P* = 0.0097). A study by Zhao et al. (21) demonstrates that DM with a history of ASCVD is associated with an increased risk (> 20%) of recurring events. Although the patients had well-controlled LDL-C levels on moderate-intensity statins, the study indicated the need for better LDL-C control and other risk factors in high-risk ASCVD populations (21). Increasing age is also significantly correlated to LDL-C goal non-achievement, as per a multicentre study conducted in Vietnam (43) and a South Korea-based study, whereby the non-achievement of the LDL-C goal was observed in older patients with an average age of 61.9 years (44). Similar observations were made in this AcarD study, wherein patients above the age of 75 years were less likely to attain the LDL-C goal of < 1.4 mmol/L (*P* = 0.0087). Therefore, the risk of ASCVD can be mitigated by early maintenance of optimal lipid levels (14). Another significant association was observed between LVEF < 35% and lower LDL-C target achievement (*P* = 0.0325). In this AcarD study, only a small proportion of patients [4.67% (*n* = 5/40)] had LVEF < 35% and targeted LDL. Hence, the correlation between LVEF < 35% and the LDL-C goal should be further explored with a larger sample.

Currently, a gap exists between guideline recommendations and real-world clinical practice. Despite a higher proportion of patients undergoing treatment with high-intensity statins, challenges prevail in achieving the LDL-C treatment goal. Clinical inertia and limited access to LLTs obstruct the optimal achievement of target goals (45). Non-adherence to LLTs is another hurdle in attaining treatment goals in patients with ASCVD (45). A multidisciplinary team, the Cardiovascular Risk Reduction Clinic (CRRC), comprising doctors, nurses, dieticians, physiotherapists, and pharmacists, was established by IJN to improve outcomes in patients with high ASCVD risk. A standardised management protocol with predefined inclusion criteria, risk stratification, a patient monitoring checklist for outcomes tracking, and patient educational materials including guideline-directed care, therapeutic lifestyle, and medication adherence counselling form the framework of CRRC. A short-term real-world outcome measurement of patients before and after recruitment into IJN CRRC has demonstrated changes in treatment patterns by optimising LLT treatment and a statistically significant proportion of patients exhibiting improved LDL-C reduction and goal achievement with CRRC (46).

Despite the range of parameters analysed in this study, there are some drawbacks. The results of this study may not be representative of the true ASCVD population due to the inclusion and exclusion criteria of this study and may not be representative of ASCVD patients nationwide, as this is a single-centre study. Moreover, data completeness and accuracy are limitations to be considered. As the data were retrieved from medical charts, there is a risk of inadequate information due to recall bias and/or poor documentation. Due to the small sample size and variability in the data, logistic regression analysis was not feasible. However, further research is warranted to investigate potential correlations between risk factors and achievement of LDL-C goals.

## Conclusion

In this study, hypertension, diabetes, followed by CKD and elevated BMI, were the highly prevalent risk factors observed among ASCVD patients with dyslipidaemia. The achievement of the LDL-C treatment goal of < 1.4 mmol/L, as per the ESC guideline, was only 24.83% at 1 year, with a high proportion unable to achieve the guideline-recommended targets. While a high proportion of patients were administered high-intensity statin monotherapy, use of combination therapies was suboptimal, despite patients with established ASCVD. Thus, combination therapy usage and innovative therapies are required to manage these patients in achieving risk-based LDL-C goals. This study provided insights into the risk factors, LLT patterns, and unmet needs among hospitalised ASCVD patients, thereby helping to understand and manage treatment strategies in the local Malaysian population and emphasising the importance of LDL-C goal achievement within key patient subgroups.

## Figures and Tables

**Table 1:** Baseline characteristics of ASCVD patients

Variables	Categories	Patients *n* (%)	Mean (SD)
Age group (years)	< 40	14 (3.25)	60.53 (9.86)
	40 to 59	179 (41.53)	
	60 to 75	215 (49.88)	
	> 75	23 (5.34)	

Gender	Female	89 (20.65)	-
	Male	342 (79.35)	-

Race	Chinese	45 (10.44)	-
	Indian	116 (26.91)	-
	Malay	265 (61.48)	-
	Others	5 (1.16)	-

Smoking history	Yes	37 (8.66)	-
	Ex-smoker	28 (6.56)	-
	No	362 (84.78)	-

LDL-C (mmol/L)	< 1.0	36 (8.35)	1.97 (0.88)
	1.0 to 1.39	60 (13.92)	
	1.4 to 1.79	103 (23.90)	
	1.8 to 2.59	145 (33.64)	
	≥ 2.6	87 (20.19)	

TC (mmol/L)	< 5.2 mmol/L	392 (90.95)	3.74 (0.97)
	5.2 to 6.2 mmol/L	32 (7.43)	
	> 6.2 mmol/L	7 (1.62)	

Types of ASCVD	MI/UA (ACS)	381 (88.40)	-
	Stable angina	101 (23.43)	
	PAD	5 (1.16)	
	Ischaemic stroke	1 (0.23)	
	TIA	1 (0.23)	
	Ischaemic stroke	1 (0.23)	

Heart rate (bpm)		403	69.84 (11.74)

Blood pressure (mmHg)	Systolic	408	132.82 (20.74)
	Diastolic	408	77.16 (11.46)

ACS = acute coronary syndrome; ASCVD = atherosclerotic cardiovascular disease; LDL-C = low-density lipoprotein cholesterol; MI = myocardial infarction; PAD = peripheral arterial disease; TIA = transient ischaemic disease; TC = total cholesterol; UA = unstable angina

**Table 2:** Proportion of ASCVD patients stratified by risk factors/comorbidities

Risk factors/comorbidities		*N* (%)
DM		224 (51.97)

AF		18 (4.18)

Multivessel CAD		122 (28.31)

LVEF < 35% at admission		40 (9.28)

CKD (≥ Stage 3a)		123 (28.54)

CKD stages	Stage 1 (eGFR ≥ 90)	95 (22.04)
	Stage 2 (eGFR 60 to 89)	213 (49.43)
	Stage 3a (eGFR 45 to 59)	62 (14.39)
	Stage 3b (eGFR 30 to 44)	37 (8.59)
	Stage 4 (eGFR 15 to 29)	10 (2.33)
	Stage 5 (eGFR < 15)	14 (3.25)

PAD		5 (1.16)

Smoking	Smokers	37 (8.66)
	Ex-smokers	28 (6.56)

Systemic hypertension		329 (76.33)

History of cardiac surgery/intervention		110 (25.52)

BMI	Underweight (< 1.85 kg/m^2^)	3 (0.71)
	Normal (18.5 to 22.9 kg/m^2^)	110 (25.94)
	Overweight (23 to 27.4 kg/m^2^)	196 (46.22)
	Obese (≥ 27.5 kg/m^2^)	115 (27.13)

Recurrent episode of ASCVD-related event in < 12 months		59 (13.69)

Young age at first ASCVD event (< 40 years)		12 (2.78)

Age > 75 years		23 (5.34)

AF = atrial fibrillation; ASCVD = atherosclerotic cardiovascular disease; BMI = body mass index; CAD = coronary artery disease; CKD = chronic kidney disease; DM = diabetes mellitus; eGFR = estimated glomerular filtration rate; LVEF = left ventricular ejection fraction; PAD = peripheral arterial disease
